# Is It Enough to Diagnose Pheochromocytoma by Measuring Urine Metanephrines Levels?

**DOI:** 10.7759/cureus.69560

**Published:** 2024-09-16

**Authors:** Mehdi Houssein, Cevdet Aydın, Ekin Yiğit Köroğlu, Abbas Ali Tam, Sevilay Sezer, Zeynep Devran, Oya Topaloglu, Reyhan Ersoy, Bekir Cakir

**Affiliations:** 1 Endocrinology and Metabolism, Ankara Bilkent City Hospital, Ankara, TUR; 2 Endocrinology, Ankara City Hospital, Ankara, TUR; 3 Endocrinology and Metabolism, Ankara Yıldırım Beyazıt University School of Medicine, Ankara, TUR; 4 Medical Biochemistry, Ankara Bilkent City Hospital, Ankara, TUR; 5 Public Health, Sakarya Provincial Health Directorate, Presidency of Public Health Services, Sakarya, TUR

**Keywords:** adrenal adenoma, pheochromocytoma, plasma free metanephrines, sensitivity, urinary metanephrines

## Abstract

This study aimed to evaluate the diagnostic value of plasma and urinary metanephrines and determine their sensitivity, specificity, and correlation among patients with adrenal lesions, which required a multidisciplinary approach because of size, functional state, or malign appearance. This retrospective study of 152 patients with adrenal lesions was conducted at the Outpatient Clinics of Ankara Bilkent City Hospital at the Department of Endocrinology and Metabolism Diseases between August 2019 and February 2022. These patients were discussed at the Endocrinology and Surgery multidisciplinary council because of their adrenal lesions. Among them, 94 patients underwent adrenal surgery. Thirty patients with histologically proven pheochromocytoma, 36 with cortical nodular hyperplasia, four cases of adrenocortical carcinoma and 24 cases with angiomyolipoma were detected. According to the analysis, the most sensitive test in diagnosing pheochromocytoma was urinary fractionated metanephrine (90%), followed by plasma free metanephrines at 84 %. Conversely, the most specific test was plasma free normetanephrine (91.4%). A statistically significant correlation exists between plasma and urinary metanephrine; plasma normetanephrine levels were also significantly correlated with urine normetanephrine. These findings indicate that plasma and urine metanephrines are sensitive for detecting pheochromocytoma and can be used interchangeably.

## Introduction

It is important to determine whether an adrenal incidentaloma is a hormonal hypersecretion lesion or not. Studies show that hypersecretion of hormones such as aldosterone, cortisol, or catecholamines is found in 12% to 23% of adrenal incidentaloma [1]. In terms of catecholamines, hypersecretion plasma free metanephrine is known to be the most sensitive screening test. Also, 24-hour urinary total metanephrine measurement can be performed as an alternative test [2].

Besides, catecholamine excess should be investigated among patients with the classic triad of palpitations, headache, and diaphoresis or in case of tremor and facial pallor. Severe hypertension with target tissue damage such as hypertrophic or dilated cardiomyopathy, increased basal metabolism rate, weight loss, sweating, heat intolerance, and altered glucose homeostasis resulting in type 2 diabetes mellitus, hypercalcemia, and polycythemia should also be examined for catecholamine excess [3].

Moreover, pheochromocytoma must always be excluded in patients with incidental adrenal nodules that have an attenuation value greater than 10 Hounsfield units, according to the last WHO adrenocortical diseases guidelines published in 2022. An aspiration biopsy may result in a catastrophic outcome in a patient with an unsuspected pheochromocytoma [4]. Thus, plasma free fractionated metanephrines or 24-hour urinary metanephrines should be measured.

Metanephrines are found to be increased in 95-97% of patients with pheochromocytoma. Measurement of metanephrine and normetanephrine concentrations in 24-hour urine is a reliable screening test. The sensitivity of urinary free metanephrines is equal to the measurement of free metanephrines in plasma (96% vs. 98%), but the specificity of urinary metanephrine is significantly lower (69%/89%) [5]. So, plasma metanephrine and normetanephrine levels are tests with very high negative predictive value, with normal plasma concentrations eliminating the diagnosis of pheochromocytoma [2]. We, in this retrospective study, examined the diagnostic efficacy and the correlation between plasma concentrations of metanephrines and normetanephrines compared with urinary excretion of metanephrines and normetanephrine among patients discussed at the endocrine-surgery multidisciplinary meeting for adrenal lesions that may require surgical intervention (risk of pheochromocytoma, hormone-producing, adenoma, adrenocortical carcinoma or size >4 cm). Patients discussed in the multidisciplinary team meeting are referred by endocrine polyclinic doctors. Criteria for referral are radiologically atypical adrenal appearance (heterogeneity, high Hounsfield Unit (HU) value, larger than 4-6 cm) and functional adenomas. A multidisciplinary team meeting includes endocrinologists and endocrine and general surgery/urology experts.

## Materials and methods

This cross-sectional retrospective study was carried out at the Outpatient Clinics of Ankara Bilkent City Hospital at the Department of Endocrinology and Metabolism Diseases between August 2019 and February 2022. Within this time period, 152 patients with adrenal lesions were admitted and discussed at the multidisciplinary expert team meeting. Patients discussed in the multidisciplinary team meeting were referred by endocrinologists from outpatient clinics. Adrenal lesions considered in our meeting may be incidentalomas large in size (>4 cm) or thought to be functional, or adrenal lesions with clinical features (sustained hypertension, paroxysmal hypertension, sustained hypertension with paroxysms, and normotension) or atypical radiological appearance (large (4-6 cm), heterogeneous masses with areas of necrosis and cystic change and density always >10 HU) risk of pheochromocytoma or adrenocortical carcinoma.

Noradrenaline, adrenaline, dopamine, free fractionated metanephrines, and normetanephrine are measured in plasma and 24-hour urine specimens or high-performance liquid chromatography-electrochemical detection (HPLC/ED) techniques. Blood samples were collected from 145 patients using a forearm venous cannula with patients in a supine position. Among the seven patients included in the study, plasma metanephrines tests were not obtained (the exact reason is not known). Patients were instructed to fast overnight, and 24-hour urine samples were collected from all patients into acid-containing bottles. No dietary precautions were given before collection. Pharmacologic Interference was excluded. An elevation of a two-fold upper reference range was accepted as strongly diagnostic of pheochromocytoma. The decision of the multidisciplinary meeting was taken based on laboratory findings, medical history, and radiological features (large lesion, mostly >4 cm, evidence of hormone excess, and evidence of significant tumor growth during follow-up imaging).

We reviewed the medical records of patients. Pathologic diagnosis, demographic data, and laboratory test results were also reviewed, especially in terms of plasma free fractionated metanephrines and 24-hour urinary metanephrines, as the diagnosis of pheochromocytomas depends mainly upon the demonstration of catecholamine excess by 24-hour metanephrines or plasma metanephrines [6]. And we examined the diagnostic efficacy and the correlation between them.

Statistical analyses were performed using SPSS software, version 16.0 (SPSS Inc., Chicago). The suitability of the variables to normal distribution was examined using visual (histogram and probability graphs) and analytical methods (Kolmogorov-Smirnov/Shapiro-Wilk tests). Descriptive analyses were performed on median and interquartile range (IQR) values as variables not normally distributed. The non normally variables distributed numerical variables were compared with the Mann-Whitney U test. Spearman’s correlation tests were used for the non-normally distributed variables.

## Results

Between 2019 and 2022, 152 patients with adrenal disease were discussed multidisciplinary, and 61.8% of patients underwent adrenalectomy. The mean age of the patients was 55.7 years; 57% were male and 42% were female. The number of patients who were considered to have pheochromocytoma according to the medical history and the laboratory results was 48 (31%). On performing the histopathologic examination, 31% (n=30) of patients were diagnosed with pheochromocytoma, 36.17% (n=34) as adenoma or cortical nodular hyperplasia, 4.25% (n=4) cases of adrenocortical carcinoma and the remaining cases (25.53%) were reported as angiomyolipoma (Table 1).

**Table 1:** Patients with a positive plasma and urine catecholamine ratio underwent surgery *Positive values were considered as two-fold above the higher reference value.

Histopathologic diagnosis	Plasma free metanephrine	Plasma free normetanephrine	Urine metanephrines	Urine normetanephrines
Pheochromocytoma	84 %	75 %	90 %	54,3 %
Cortical Adenoma	2 %	3 %	1 %	8 %
Angiomyolipoma	0 %	3 %	1 %	13 %
Adrenocortical Carcinoma	0 %	0 %	0 %	0 %

Of the 30 patients with pheochromocytoma on final pathologic analysis, plasma free metanephrine and normetanephrine were measured in 23 patients. Nineteen (84%) patients had a diagnostic elevation in plasma metanephrine levels, and 17 (75%) in plasma normetanephrine levels (two-fold above the normal range). Urinary fractionated metanephrines and normetanephrine measurements were performed in all cases of pheochromocytoma; their concentrations were found to be diagnostically significant in 27 (90%) and 16 (54.3%) patients, respectively (two-fold above the normal range). Although diagnosed with pheochromocytoma, plasma free metanephrine was normal in four patients, and urine metanephrine was within normal limits in three cases (Table 2).

**Table 2:** Sensitivity, specificity, false positive and false negative rates of plasma and urine catecholamines

Catecholamines metabolites	Sensitivity	Specificity	False positivity	False negativity
Pasma free metanephrine	84.6%	82.6%	15.4%	17.4%
Plasma free normetanephrine	75%	91.4%	25%	8.6%
Urine metanephrines	90%	84.3%	10%	15.7%
Urine normetanephrines	54.3%	90.9%	45.7%	9.1%

Among other patients in whom pheochromocytoma was excluded based on histology, pre-surgery plasma free metanephrine levels were high in only 2% of patients with pathology is compatible with cortical adenoma. In the remaining cases (angiomyolipoma, adrenocortical carcinoma) plasma free metanephrine levels were within the normal range. Plasma free normetanephrine level was found to be high in 3% of cortical adenoma and 3% of angiomyolipoma patients. Urinary fractionated metanephrine was found to be high in 1% of cortical adenomas and 1% of angiomyolipoma cases, whereas urinary fractionated normetanephrine was high in 8% of adenomas and 13% of angiomyolipoma cases. A remarkable point is that in all ACC cases, both plasma and urine catecholamines were within their normal limits (Table 2).

For all patients with pheochromocytoma, sensitivities were the highest for measurements of urinary fractionated metanephrines at 90%, followed by plasma free metanephrines at 84%. Sensitivities of both the above tests exceeded those for plasma free normetanephrine (75%) and urinary fractionated nometanephrines (54.3%). Plasma free metanephrine and normetanephrine yielded a specificity of 84.6% and 91.4 % for the detection of pheochromocytoma. Urinary metanephrine and normetanephrine had a specificity of 84.6% and 90.9% respectively. Therefore the most sensitive test in the diagnosis of pheochromocytoma was urinary fractionated metanephrine, while the most specific test was plasma free normetanephrine (Table 3). Moreover, there were significant correlations between plasma and urinary metanephrines (p<0.01). In addition, plasma free normetanephrine levels were significantly correlated with urine normetanephrine (p<0.01) (Table 3).The false positive rate of urine fractionated normetanephrine was the highest (45.7%).

**Table 3:** Correlation coefficient between plasma and urine catecholamines r: correlation coefficient; p<0. 005 is considered highly statistically significant.

Catecholamines metabolites	Pheochromocytoma	Non- Pheochromocytoma
Plasma and urine metanephrine	r = 0.7	r = 0,48
	p <0.001	p <0.001
Plasma and urine normetanephrine	r = 0.565	r = 0.497
	p<0.005	p< 0.001

Considering the plasma metanephrine measurements as the reference test and urinary metanephrine as an index test, the area under the curve in the receiver operating characteristic curve (ROC) analysis is 89.3% (statistically significant). An optimal cut-off value of 172.9 mcg/24 hours maximizes sensitivity (94%) (Table 4).

**Table 4:** Sensitivity and specificity values ​​according to the cut-off values of urinary metanephrines

Cut-off (urinary metanephrine)	Sensitivity (%100)	Specificity (%100)
167	94	67
172	94	68
179	82	68
181	82	69
197	76	72
200	76	74
208	76	77
216	76	78
245	76	80

## Discussion

Although biochemical diagnosis of pheochromocytoma has always been a subject of research for many years, it is still an attractive area because of the unanswered questions about the more accurate tool for diagnosis or for exclusion among patients with adrenal lesions or patients with high suspicion. To date and for this purpose, most centers use urine metanephrine levels as plasma metanephrine measurements are not widely available, but is it enough?

Detecting pheochromocytoma is too crucial to avoid the potentially serious consequences of non-treatment, so the most sensitive test to establish the diagnosis remains a matter of debate. Some researchers have concluded that plasma metanephrines are more sensitive in the detection of pheochromocytoma compared with urinary measurements [6,7]. Others have reported that urinary catecholamines and their derivatives have higher sensitivities than plasma measurements [8,9]. In our study, we found that urinary fractionated metanephrines were the most sensitive diagnostic test, followed by plasma free metanephrines. We also showed that plasma normetanephrine testing has the highest specificity. Although the latter is different from previous studies [10,11], it is actually not important because pheochromocytomas are life-threatening without treatment. Hence, the main purpose is to define the most sensitive test that can exclude false-negative results.

According to the literature, plasma metanephrines measurement is superior to urine measurements. The sensitivity of plasma metanephrines measurement is 89.5-100%, and its specificity is 79.4-97.6%, while the sensitivity of urine metanephrines measurement is 85.7-97.1% and its specificity is 68.6-95.1% [10,11], but despite this fact, 24-hour urinary fractionated metanephrines are widely used as the first test for suspicion cases of pheochromocytomas. We basically intended to assess and compare the results of both plasma and urine metanephrines, and we found that both results were correlated in histopathologically confirmed pheochromocytoma cases and also among non-pheochromocytoma operated adrenal lesions. These results reinforce that both tests can be used as alternatives to each other in the diagnosis of pheochromocytoma.

Although measurements of normetanephrine and metanephrine provide a highly sensitive test for diagnosing pheochromocytoma, false-positive results remain a problem. That is why, before biochemical tests, some consideration should be given to possible causes of false-positive results, especially medications, inappropriate sampling conditions, and diet. In our study, there were no drugs that could interfere. Blood and urine samples were collected after an overnight fast. In our study, the urine normetanephrine was found to have the highest false positive value. This could be attributed to several factors that may cause an increase in catecholamines and metabolites, such as serious physical stresses, contrast agents, or caffeine and black tea. Conversely, false negatives aren't common, which is the case in our study, but they can occur among patients with small or microscopic tumors (1 cm) that produce only small amounts of catecholamines and others that do not produce norepinephrine or epinephrine.

‘’Phaeochromocytoma-a laboratory experience’’ [12], "Laboratory diagnosis of phaeochromocytoma: which analytes should we measure?’’ [13], ‘’Laboratory contribution to the diagnosis of pheochromocytoma’’ [14] and many other similar studies has been performed and is still being, but although pheochromocytoma continue to be a challenge as there are small tumors which do not release catecholamines, and others that only produce dopamine , and as we mentioned above false-positive results are common for both plasma free and urine fractionated metanephrines. Besides, and unfortunately, normal values do not exclude pheochromocytoma, henceforth, medical history, radiologic and functional imaging, genetic testing, and sometimes chromogranin may be required [15-17].

Our study supports the utility of urinary metanephrine as the most sensitive test in the detection of pheochromocytoma followed by plasma free metanephrine. This feature can be considered an additional advantage in areas where plasma metanephrine levels cannot be measured. The strength of our study is that it includes patients discussed in a multidisciplinary expert meeting with histologically confirmed pheochromocytoma. More importantly, it shows the diagnostic power of urine metanephrine confirmed by ROC analysis. Limitations include the retrospective nature of the study and the relatively small number of patients.

## Conclusions

Plasma and urine metanephrine have the most important role in the diagnosis and exclusion of pheochro mocytoma and can be used as alternatives to each other.
